# Seeking menstrual products: a qualitative exploration of the unmet menstrual needs of individuals experiencing homelessness in New York City

**DOI:** 10.1186/s12978-021-01133-8

**Published:** 2021-04-13

**Authors:** Caitlin Gruer, Kim Hopper, Rachel Clark Smith, Erin Kelly, Andrew Maroko, Marni Sommer

**Affiliations:** 1 Department of Sociomedical Sciences, Mailman School of Public Health, Columbia University, 722 W. 168th Street, Room 537, New York, NY 10032 USA; 2 Department of Environmental, Occupational, and Geospatial Health Sciences, CUNY School of Public Health, 55 W. 125th Street, Room 508, New York, NY 10027 USA

**Keywords:** Menstruation, Homelessness, New York City, Menstrual product

## Abstract

**Background:**

There has been increasing recognition that certain vulnerable populations in the United States of America struggle to meet their menstruation-related needs, including people experiencing homelessness. Media and policy attention on this subject has focused on the provision of free menstrual products to vulnerable populations, including a New York City legislative bill, which guarantees access to menstrual products for Department of Homeless Services shelter residents (Intros 1123-A).

**Methods:**

This qualitative study explored the challenges people experiencing homelessness in New York City face in accessing menstrual products. Data collection was conducted from June to August 2019 and included: Semi-structured key informant interviews with staff from relevant government agencies and homeless service providers (n = 15), and semi-structured in-depth interviews with individuals with experience living on the street and in shelters (n = 22). Data were analysed using thematic analysis.

**Results:**

Key themes that emerged included: (1) insufficient and inconsistent access to menstrual products; (2) systemic challenges to providing menstrual products; and (3) creative solutions to promote access to menstrual products. Both shelter- and street-living individuals reported significant barriers to accessing menstrual products. While both populations struggle, those in shelters were more likely to be able to purchase menstrual products or access free products at their shelter, while those living on the streets were more likely to have to resort to panhandling, theft, or using makeshift materials in place of menstrual products. Across both populations, individuals described barriers to accessing free products at shelters and service providers, primarily due to distribution systems that rely on gatekeepers to provide a few pads or tampons at a time, sometimes of inadequate quality and only upon request. Shelters and service providers also described challenges providing these products, including inconsistent supply.

**Conclusion:**

These findings highlight the critical importance of expanding and improving initiatives seeking to provide access to menstrual products for vulnerable populations. Despite policy level efforts to support menstrual product access, individuals experiencing homelessness in New York City, whether living in shelters or on the street, are often not able to access the menstrual products that they need to manage their monthly menstrual flow.

## Plain English summary

In recent years, the media and policymakers have drawn attention to the challenges that people experiencing homelessness in the United States face managing their menstruation. There are a growing number of initiatives focused on providing menstrual products (pads, tampons, etc.), including a bill passed in New York City requiring homeless shelters to provide these items to clients. This study explores the menstruation experiences of people with experience living in shelters or on the streets of New York City. Qualitative data was gathered through interviews with 22 individuals with experience of homelessness, and 15 staff from relevant government agencies and service providers. Data were analysed using thematic analysis. Three main themes were found: challenges individuals experiencing homelessness faced accessing menstrual products; difficulties shelters, and service providers experienced providing access to these products; and recommendations for improvements. All participants described difficulty obtaining sufficient menstrual products to manage their monthly period. People living in shelters could more often purchase the products themselves, while those living on the streets sometimes had to resort to theft, panhandling or makeshift alternatives. Participants also described challenges accessing free products at shelters and service providers, because of the distribution methods, while shelter and service provider staff reported difficulties maintaining consistent stock. Recommendations include: increasing free product distribution in locations open twenty-four hours a day; training shelter and service provider staff to improve their comfort providing these services; and rethinking distribution strategies. Our findings emphasize the importance of initiatives that provide access to these essential items for vulnerable populations.

## Background

Menstruation has gained global attention in the past decade as a critical public health and human rights issue [1–3]. This includes growing evidence documenting what those who menstruate need in order to manage their monthly periods comfortably and confidently: access to safe, private toilets with adequate water supply, bathing and laundering spaces, menstrual materials and supplies (e.g. menstrual products, underwear), and quality information and education [4–9]. While attention initially focused on low-income countries, there has been increasing recognition that certain vulnerable populations in the United States of America (USA) also struggle to meet their menstruation-related needs [10, 11]. For instance, people experiencing homelessness face unique challenges to managing their menstruation whether living in shelters or on the street [12, 13], such as lack of access to menstrual products [14–16], difficulty maintaining personal hygiene [17], menstrual shame and stigma [18, 19], and gynaecological issues [20]. This paper seeks to add further nuance to this picture of unmet need by exploring how those experiencing homelessness in New York City (NYC) manage their periods, and how—short of re-housing—their needs could be more effectively addressed. We focus in particular on difficulties related to securing menstrual products.

NYC has the largest population of people experiencing homelessness in the USA. In 2019, an average of 63,839 people slept in NYC homeless shelters every night [21], and a point-in-time count estimated that at minimum an additional 3,588 individuals were living on the streets [22]. The latter is likely much higher due to difficulties in locating and estimating this population [23]. The number of women in the shelter system has continued to increase over the last 18 years [21, 22]. Additionally, and likely an underestimate, the Department of Housing and Urban Development’s (HUD) 2018 point-in-time estimate of homelessness suggested that 0.7% of those experiencing homelessness identify as transgender or gender-nonconforming [24]. All told, the number of people menstruating while experiencing homelessness in NYC is significant.

Existing evidence suggests that this population encounters substantial difficulties related to menstruation, including acquiring menstrual products, accessing bathrooms, and maintaining basic personal hygiene [14, 19]. These challenges are compounded by the stigma still attached to menstruation, marking it as something to be hidden from view and shielded from others [25]. Societal norms stipulate that its management be discreet and secret. For people experiencing homelessness, this is particularly challenging: they often lack access to the resources required to avoid leaks and prevent menstrual accidents, thus reducing their chances of maintaining their appearance and blending in with the general public. Perversely, this ability to “pass” as someone conventionally housed plays a vital role in their ability to access precisely those resources—commercial bathrooms, employment opportunities—that would better equip them to manage their menstruation [26, 27].

Access to menstrual products has received much attention from the media and policymakers. In 2016, NYC passed a legislative bill which guarantees access to menstrual products for Department of Homeless Services shelter residents (Intros 1123-A). Referred to as the *NYC Menstrual Policy* for the purposes of this paper, Intros 1123-A requires city-run and city-funded shelters to provide tampons and pads to clients. It applies to family shelters, single adult shelters, HIV/AIDS shelters, domestic violence shelters, youth detention facilities, and congregate care facilities for youth waiting for placement with a foster care agency [28]. While some shelters provided these products already, the bill sought to codify this service, ensuring that all shelter residents have a source of these necessities. This bill was the first of its kind in the USA, though since its passing, other cities and states, such as Los Angeles, Chicago, and New York State, have instituted similar policies [10].

This study was conducted in NYC between May and August 2019 in collaboration with the Coalition for the Homeless, one of the oldest advocacy and direct service organizations in NYC. The Coalition provided invaluable insights into the predicament of those facing homelessness in NYC today, and facilitated the connection to individuals experiencing homelessness and to service providers throughout the city.

## Methods

We conducted a qualitative study that sought to understand the menstruation experiences of people living on the streets and in shelters in NYC. The research methods included: (1) key informant interviews (KII) conducted with staff from relevant government agencies and homeless service providers; and (2) in-depth interviews (IDI) with people experiencing homelessness while menstruating. This paper will explore findings from the KIIs and IDIs related to menstrual products; other findings from the KIIs and IDIs are described elsewhere [27].

The study received Internal Review Board (IRB) approval from the Columbia University Institutional Review Board*.*

### Sample and recruitment

The sample for the KIIs included a range of government and service provider actors (male and female) from agencies and organizations providing services to people experiencing homelessness. They included both frontline providers, and those with relevant oversight or management roles. Key informants (n = 15) were sampled purposively to ensure that participants represented agencies and organizations conversant with the issue, existing challenges, and/or recommendations for possible improvements to the current situation. This included relevant government agencies, food pantries, youth shelters and street outreach providers.

The sample for the IDIs included people with experience living on the street and/or in shelters ages 18 years and older. To capture a diversity of menstruation-related issues, we sought to interview individuals across a range of age categories including: 18–25; 26–35; 36–45; and 46 and above. IDI participants (n = 22) were identified from among those seeking services at homeless service providers using convenience sampling. These service providers provided clients with information about the study via announcements, posted flyers, and sign-up sheets at the check-in desk. Interested clients identified themselves to the service provider who in turn introduced them to the research team. IDI participants received a small incentive for their participation ($10 or $15 subway card).

All participants presented as female; however, we did not explicitly ask about gender identification. The age of the participants ranged from 18 to 62, and all participants had menstruated while experiencing homelessness. All participants had lived in a shelter, and a segment (n = 7) of the participants had experienced both street and shelter living.

### Data collection

KIIs and IDIs were conducted in NYC by two-person research teams during the period of June–August 2019. The research team consisted of the Principal Investigator (PI), a graduate research assistant (RA), and a member of the PI’s staff research team. All interviewers were females and had conducted work or research with individuals experiencing homelessness in the past. Before starting data collection, the team conducted reflexivity exercises to consider our positionality and the power dynamics in the exchange, as well as discussing own experiences menstruating while living in NYC. The larger research team also included a Co-investigator (Co-I) with significant experience in research, practice and policy related to homelessness. All participants provided informed consent prior to the start of each interview.

Based on respondent preference, KIIs were conducted in person or online. The interviews ranged from 30 to 60 min, were conducted in a confidential setting, and were digitally recorded unless otherwise requested by the interviewee. We used a semi-structured interview guide tailored to the organization’s work and mission, its role in providing support to people experiencing homelessness, the specific needs and challenges encountered while providing this support (related to menstruation and more broadly), and any recommendations for how these experiences could be improved.

IDIs were conducted at the offices of the collaborating service providers in a confidential space where the conversation could not be overheard and were digitally recorded with the consent of the participant. The interviews were conducted using a semi-structured interview guide that explored the participant’s current and recent housing status and personal history, before delving into the participant’s experience managing their menstruation while experiencing homelessness. This included questions around the facilities and alternate locations used as bathrooms, bathing and laundering; access to menstrual products; and their recommendations for improving the menstrual experiences of people experiencing homelessness.

### Data analysis

Interviews were transcribed and analysed by two members of the research team, using Malterud’s ‘systematic text condensation’ method for thematic cross-case analysis [29]. This included the following steps: (1) identification of preliminary themes; ((2) creative development of qualitative codes; (3) condensation of coded text; (4) synthesis and reconceptualization [29]. The PI reviewed and helped to revise the final codebook, and the analysis team used Dedoose analytic software to code the data. The themes identified from the data were shared with full research team for discussion, refinement, and validation. The full team met multiple times to discuss the themes identified, the commonalities and differences emerging within the themes, and to discuss and resolve discrepancies between codes. After data analysis was complete, the final analytical themes were presented to representatives from the Coalition for the Homeless for discussion and to affirm the data. The resultant analytical themes are presented below, along with illustrative excerpts from interviews.

## Results

Three key themes related to the availability and accessibility of menstrual products for people experiencing homelessness in NYC proved salient throughout the analysis: (1) insufficient and inconsistent access to menstrual products; (2) systemic challenges to providing menstrual products; and (3) creative solutions to promote access to menstrual products.

### Insufficient and inconsistent access to menstrual products

An overarching finding from respondents with experience living both in shelters and on the street was the formidable struggle to access menstrual products consistently. Those living in shelters most commonly reported purchasing their own menstrual products, often relying on discount or dollar stores to minimize costs. Revenue sources varied, with a majority of participants reporting using their own income, depending on financial support from friends or family members, or public assistance funds. Trade-offs made with respect to other necessities were common. For example, securing menstrual products could mean going without medications or more expensive food items. Others reported having to rely on makeshift solutions when they were unable to afford products:

*I would run out of money and I ended up doing things rather than [buying them]… like, cutting up old t-shirts and stuff like that.*—IDI 15

Respondents living in shelters also mentioned shelter-based distributions, though less frequently. Depending on the shelter, the distribution method varied: mass distributions on designated supply days; requests to a caseworker, shelter staff, or the check-in desk; or, rarely, unencumbered access to a centrally located repository. A few participants reported that such distributions made their lives easier by reducing their financial burden and ensuring that they could access products more regularly. Consistently, though, participants described the availability of products as uneven and insufficient. Some reported a complete absence of menstrual products at their shelter.

Rationing methods, too, were described, with menstrual products being distributed by staff members selectively and only upon request. Participants reported receiving only a few pads or tampons at a time, necessitating asking for products repeatedly, sometimes multiple times a day. Some shelters permit product requests only on specific distribution days during a given week or month; still others require the request be made to a certain staff member who is only on duty during certain days or hours. Such restrictive distribution systems meant that—even where officially “provisioned”—respondents could not rely on shelters as a primary source of products.

A number of shelter clients added nuance to such requests, describing how disclosing their need for menstrual products elicited shame and humiliation, feelings heightened if the gatekeeper was stationed in a public place (e.g., the check-in desk), or male:

*…the humiliation is that you have to keep going back to them and asking them, and when you're asking for, because they have police and security, so it's not private. So you're asking for it in front of NYPD and DHS security, and most of those are male staff.* —IDI 07

Other shelter clients were simply unwilling to disclose their menstruating status due to social norms and taboos, preventing them from accessing the free menstrual products even when available. A few respondents reported experiencing or witnessing harassment, including sexual harassment, by shelter staff:

*… the women have to go and ask for menstrual products, and then you have the male staff smiling, smirking at them… Women feel that they're being discriminated against, harassed, sexually harassed because they're menstruating* —IDI 07

The types and quality of menstrual products provided by the shelters were also identified as problematic. For example, some women preferred using tampons but only pads were available, or vice versa. This meant settling for what was available, or finding another way to acquire their preferred product. Many complained about the quality or absorbency of available products, saying that pads provided by their shelter were too small or too thin to manage what they described as their “heavy [blood] flow.” This made it necessary to change the product much more often, requiring access to safe, clean spaces for changing—a contingency not readily available to them [27]. As a result, even when menstrual products were available at their shelter, some participants opted to acquire their products elsewhere:

*I was always like, if I had like, six, five dollars, I'd go get pads with wings, cause it would just make life so much easier. And the reason I needed so many of those from [service provider] was cause they didn't work and I had to go to the bathroom every two three hours instead of every four hours or five.* —IDI 13

Lastly, a number of participants reported being unaware of whether their shelter provided free menstrual products or not. A few explained that it had never occurred to them that these products might be available, when so many other basic items are not, and so they had never asked. Despite clearly outlining how necessary menstrual products were for their daily life, the women did not view them as something they could expect to receive. Similarly, when questioned, most participants had not heard of the 2016 law requiring shelters to provide menstrual products, and were unaware of their legal right to access products at their shelter.

Unsurprisingly, participants with experience living on the street described even greater difficulties accessing menstrual products, as they lack access to shelter supplies and were less likely to report alternative sources of funding and support. Most commonly, these participants relied on service organizations for handouts. However, not all homeless service providers in NYC distribute menstrual products, and even when they did, availability was uncertain. Menstrual products are not viewed as an essential item in “care packages”:

*They give you a care package; they don't necessarily have personal things like that, like tampons and stuff like that. They might have deodorant and soap and toothpaste, but they don't have the personal items that you might need. So, you have to ask for that. And if they have it, they don't mind. A lot of times they don't carry it, though, so you have to go from place to place to place, and you have to go in public restrooms and stuff like that, because that's not something that we think about. And you think about giving people water and food and soap to be clean and, but you don't think about the other aspects, like vitamins, you know, and tampons and this stuff, it's, it's not so easy.* —IDI 21

Those with experience living on the street also described similar complaints heard from shelter residents with respect to procuring preferred products, settling for inferior quality and quantity, and having to deal with gatekeepers. As a result, some participants who had lived on the street reported resorting to panhandling for menstrual products, which posed its own difficulties. As one woman described: “I get more cigarettes than I could get tampons [while begging on the subway].” (IDI 16) A few participants had resorted to pilfered menstrual products while others, like their counterparts in the shelters, made do with makeshift materials, such as old pieces of clothing, or wads of toilet paper.

Although none of our participants self-identified as trans masculine or gender non-conforming, a number of key informants noted that such clients often encounter even more significant barriers to obtaining menstrual products. As one youth services provider shared:

*They're, like, presenting with . . . a gender that doesn't match someone who would be having a period, and so that's super complicated and embarrassing, um, and so I think there's just, yeah, there's so much shame and stigma around it...* —KII 05

As a result, transgender and gender non-conforming individuals may only be willing or able to access menstrual products from service providers where they feel accepted and have developed trusting relationships.

### Systemic barriers to providing menstrual products

In addition to the difficulties that participants themselves communicated in accessing menstrual products, shelter staff and service providers described how the system itself inhibits their ability to consistently provide menstrual products to their clients. While all the service providers articulated the importance of menstrual products, many described not always being able to provide their clients with regular access.

Shelters are included under the mandate of the 2016 *NYC Menstrual Policy*, which means that the Department of Citywide Administrative Services (DCAS) must provide a supply of menstrual products sufficient to meet the needs of their residents. However, shelter staff reported that the products were not always delivered in a timely enough manner to ensure a consistent stock. These delays contributed to occasional stock outs and to fear that the products might run out. In addition, awareness of the *NYC Menstrual Policy* was uneven among the shelter staff. Some knew nothing about the policy; others were uncertain about how it would be implemented over time. As the director of one shelter shared:

*I didn't know about the, the menstrual [policy], I had no idea. Cause I, in shelter, I've been working in shelter for, since 2010, um, and it was always something that we just provided to the clients, like we had to, yeah. —KII 012*

The fear of stock outs appeared to intersect with the distribution system in many shelters, as gatekeeping staff monitor the usage and supply of these products and prevent one client from taking the entire supply. Shelter staff seemed unaware of the potential stress that the rationing system may cause some clients.

Non-shelter service providers faced tougher challenges because they supply these products at their own discretion and expense. Most rely on product donations through arrangements ranging from formal partnerships with commercial companies to ad hoc donations from private individuals. While such approaches reduce procurement costs, they also make it harder for service providers to control the type and quality of products made available—an eventuality not lost on clients:

*No. No no no no no no no no no, there's no choice. You get what they've got. Because most of their stuff is donated too. Some, you have to get what they've got and utilize what they have. Sometimes they have tampons, most of the time they don't have tampons. Most of the time they have, uh, the pads. Yeah. And uh, you have to just take what they've got.* —IDI 21

Nor were service providers always able to predict when donations will arrive, which could lead to stock-outs or rationing of the products similar to what occurs within the shelter system:

*Yeah, I, yeah, I've noticed, [stock out of a certain type of product] doesn't happen a lot, but it does, you know, in any social service agency, like, it kind of fluctuates. —KII 06*

A number of key informants also described how well-intentioned donation efforts, often the most expedient source for products, did not always align with client preferences. For example, some menstrual cup companies have robust donation programs; however, to date, these products have not been popular with the clients and may be difficult for the clients to change, clean and store while living in a shelter or on the street:

*We've been partnering with [menstrual cup company] for a few years, um, they send us a big box of [menstrual cups] every 6 months or so. Um, but with the [menstrual cups] it hasn't been, it just hasn't picked up for the young people and so we've haven't gotten a shipment in the last six months since we have a big supply of it still.* —KII 10

Service providers may also face challenges with staff comfort distributing products—the reciprocal version of the “gatekeeper” issue mentioned earlier. While our sample of service provider informants was predominantly women, a few mused that their male counterparts might feel uncomfortable discussing menstruation or feel embarrassed when providing these supplies to women and transgender clients.

*I guess like one thing I was thinking about was sort of the, the diversity and gender diversity of our staff and, you know, the comfortability of asking for products or talking about problems with male versus female versus transgender staff…or even, just like how our, kind of our male staff feel [inaudible] feel about offering, um, [menstrual products].* —KII 09

A staff member’s own personal discomfort with distributing products may in turn contribute to creating an uncomfortable environment for clients.

### Creative solutions to promote access to menstrual products

The participants experiencing homelessness and those seeking to meet their needs shared a number of ideas for improving access to menstrual products. Resoundingly, the most common recommendation stressed the need to increase the availability of free and low-cost menstrual products throughout the city. To accomplish this, multiple service providers suggested system-level changes, such as the inclusion of menstrual products in public assistance programs such as Supplemental Nutrition Assistance Program (SNAP), Special Supplemental Nutrition Program for Women, Infants, and Children (WIC), or Medicare/Medicaid. As one Director of Social Services suggested:

*Most people who are like, street homeless, they're entitled to some type of medical insurance, right? I think that Advil and um, pads or tampons, whatever they want to use, should be part of like, some type of like, your health coverage. Cause it is, like, health, like, we women, we, I mean, I wish I didn't get my period, right, but unfortunately it's, we have to, right? So it's like, if they, if they have Medicaid, then I think Medicaid should be, you know, having like, maybe every month you should get like two packs of like tampons or be entitled to purchase two packs of, um, menstruation, or the menstruation cups should be something that your health insurance covers, uh, you know?* —KII 007

While some programs are constrained in what they can currently offer (e.g., SNAP and WIC cannot cover non-food items), these suggestions illustrate how these products could be incorporated into existing systems.

Two service providers and one participant living in a shelter suggested a citywide campaign to distribute free menstrual products in the same manner as NYC condoms, which are widely available and easily accessible. The equating of condoms with menstrual products, the latter an essential gendered item, pointedly raises an important issue of societal equity.

A second set of recommendations built on the idea of increased accessibility, proposing an expanded number of locations where menstrual products could be made available, especially ones accessible twenty-four hours a day. This was particularly important to participants who had spent time living on the street to ensure that they could access products during hours when service providers were closed. Proposed locations varied, but a common suggestion was public restrooms. As one participant experiencing homelessness (IDI 10) described, “I'd have [menstrual products] for free in the bathroom…[in] any public bathroom*.*”

Another participant without a stable home recommended a van that would drive around the city to certain drop-off points, distributing products to those who need them. Still another participant who had spent time living on the street recommended the installation of menstrual product vending machines across the city where products could be purchased using a credit card, or accessed by those on public assistance who would be given a specific card for the machines. Importantly, such a machine would be accessible 24 hours a day. Participants noted that these approaches would not only improve menstrual product access for people experiencing homelessness, but also for residents struggling to make ends meet and for anyone finding themselves in urgent need of a menstrual product while in the city.

Participants also had a range of suggestions for how to improve the accessibility of these products within the shelters and at other service providers. One idea was for more service providers to stock menstrual products, making it easier for clients to access products at familiar venues throughout the city. Another centred on improving the current distribution systems. The most common recommendation was that products be made freely available, instead of requiring interaction with a gatekeeper. A few participants described how this system is already in place in some shelters or service providers and how this makes accessing products less fraught:

*I would recommend that's really nice is, is what they do at, it's an awful place, [shelter], is, is, the having them available in a way so that…[you] don't even have to ask, I mean you've got women from all different types of backgrounds, some people might, some people might be shy about it. —IDI 15*

Beyond making products freely available, participants recommended smaller adjustments to improve the current systems. The most common suggestion was that a larger number of products should be distributed at one time per client, reducing the need to resupply during one menstrual period, improving clients’ sense of independence and dignity, and assuring not being “tied” to a given provider or location during their period. Plainly, too, this would increase their ability to seek other services, work or go about their day. Related, a few participants suggested that products be distributed regularly and routinely in a shelter instead of only on demand, so that clients could have consistent access without having to ask for them. There was also a repeated recommendation that service providers distribute menstruation-supportive supplies along with the menstrual products, such as soap, towels, feminine wipes, and underwear. These supplies are already available at some service providers, and are a much-appreciated resource:

*I honestly would make sure that every single shelter, because they do have the resources, that they would give any woman, any woman that's menstruating, whatever, access to what they need, how many they need, whenever. Not giving them a limit, because the human body changes. Um, access to towels, soap, and everything you can clean yourself* —IDI 20

Others recommended making sure more clients knew that menstrual products were available, given repeated indications that shelter clients often have no idea these products were stocked. Clients could receive a list of what supplies and materials are available for them, and where and how to access them. Alternatively, a list could be publicly posted detailing the supplies that are available and where they are located.

*…letting people know, this item is available to you, I think would be a huge help because people, again, you're not just gonna, if I'm always asking you for something, like you said, and they say no, then it's like, it makes me feel like when I really need something, you're definitely not gonna have it, so I feel like, even allowing people to know that these options are available for you if necessary, you know, if needed…it would be very helpful, or even like, if you go into shelter systems, and it's your first time where you've been there, they'll be like hey, and by the way, just in case needed, or even if they put like a little poster or something, letting people know that these items are available to you. —IDI 09*

Across all the interviews, the principal recommendation was, as one service provider, who had previously experienced homelessness, succinctly put: “[what we need is] just more access, in more spaces, with less stigma.” —KII 4.

## Discussion

The findings of this study uncovered multiple challenges that people experiencing homelessness, in shelters and on the street, face accessing menstrual products. Some differences did emerge: those in shelters were more likely to have a source of funds to purchase menstrual products or access to free products, while those living on the streets were more likely to have to resort to panhandling, theft, or using makeshift materials in place of menstrual products. Across both populations, while individuals were grateful that some service providers and shelters provide free menstrual products, they described many barriers to accessing these products, primarily due to a system that allots only a few pads or tampons at a time, insufficient for an entire menstrual period, and only upon request. There was a palpable sense of frustration amongst many respondents that they had to worry about how to access such a basic necessity.

Put most plainly, participants experiencing homelessness in NYC were not always able to access the menstrual products that they need to manage their monthly menstrual flow. Similar challenges have been documented in other cities across the USA. For example, a series of interview and focus group discussions with 20 adolescent women experiencing homelessness in Seattle, Washington found that the women struggled to access menstrual products, resulting in theft, or having to go without food [14]. In Albany, NY, interviews with five women experiencing homelessness revealed difficulty obtaining menstrual products from service providers and food pantries as they were frequently out of stock [15]. These populations also face concurrent challenges accessing private, safe spaces for changing menstrual products, and for bathing and laundering [16, 19, 27]. Thus, quality, high absorbency menstrual products may be particularly important in that they reduce the frequency in which an individual must find a safe, private place to change [16]. Given the challenges accessing bathing and laundry spaces, menstrual stains caused by leaking or inadequate menstrual products may be harder to resolve, requiring the wearing of clothes with visible menstrual stains [18, 27]. Beyond the embarrassment and shame that this may cause, it also impacts an individual’s ability to pass as someone who is not experiencing homelessness, which is critical for their ability to work, to move about the city without harassment, or to access commercial toilets [18, 27]. While access to quality menstrual products is by no means a solution to these problems, it is a step towards comprehensively addressing these basic needs. Just as critical, is ensuring consistent access to toilets and bathing and laundry facilities, as explored in a previous paper resulting from this study [27].

Participants shared that even when menstrual products were available through their shelter or other service providers, there were significant barriers to accessing and using these products, such as the necessity of disclosing one’s menstruating status to gatekeepers, the lack of availability of preferred type of menstrual product, and insufficient quality and quantity of products. Similar difficulties have been reported elsewhere. For example, during interviews with 40 women experiencing homelessness in Bristol, England, the women described challenges accessing menstrual products, lack of awareness about which service providers might supply them, and embarrassment around having to ask staff for products [19]. This suggests a gendered discrimination within shelter systems; an occurrence possibly rooted in an internalized misogyny that limits recognition of women’s basic needs [30].

It is important to note that individuals experiencing homelessness in NYC likely have greater access to menstrual products than most. Alone among USA cities, NYC has a court-enforced right to shelter and, while barriers remain, the *NYC Menstrual Policy* does provide a channel through which the majority of individuals experiencing homelessness in the city can (in theory) access menstrual products. In most other locales, there is no such guaranteed channel. Instead, individuals experiencing homelessness must find their own methods of acquiring these essential items. While NYC has the largest population of people experiencing homelessness in the country, it only represents a fraction of the estimated 568,000 people in the USA experiencing homelessness on a given night [31], a number that is likely on the rise due to the economic fallout of the COVID-19 pandemic. This issue has been long ignored due in part to the discomfort and socially sanctioned silence around this issue, with a pervasive assumption of personal responsibility for menstruation regardless of personal circumstance. Simply recognizing the current situation as an untenable state of affairs is a categorical break with the past.

Our findings from NYC support the benefit of the *NYC Menstrual Policy* as they reinforce that many people experiencing homelessness struggle to access menstrual products. However, the findings also highlight ways in which the current system could better meet the needs of this population. As recommended by the study participants, menstrual products should be provided through an open distribution system by removing (or reducing) the requirement that clients interact with a gatekeeper to access these products. There is anecdotal evidence that open distribution systems are viable as they are already in use in some shelters and service providers in NYC. Other small improvements could be made such as increasing the number of menstrual products provided at one time; providing a range of types of products (e.g. pads, tampons) and absorbencies; and ensuring the products are of adequate quality. In addition, expanding the mandate and associated resources to a broader array of providers of homeless services could make it easier for individuals living on the street to access these products. Currently, it is easier for an individual experiencing homelessness to obtain soap, a toothbrush, or toothpaste, than it is to access menstrual products; however, menstrual products are just as essential.

Policies could be considered to promote menstrual product access for all populations across the city, such as the wide-scale distribution of menstrual products modelled on the NYC condom campaign suggested by our participants, or the recent Scottish legislation mandating free menstrual products in all public spaces and for those in need [32]. There are a few examples of these types of broader initiatives appearing around the USA [33]; however, to date, the only ones that have passed and been implemented have been small-scale pilots in small, wealthy localities, where the demand for these products is likely quite low [34].

All policies or programs that seek to address the menstrual challenges faced by individuals experiencing homelessness must consider the way that menstrual stigma may impact the delivery and uptake of services. As mentioned above, systems that require little to no interaction with a gatekeeper are preferable. Service providers should consider the potential gendered inequity inherent in the notion that these resources require a gatekeeper, especially if other necessities are freely available. Additionally, all shelter and service provider staff might benefit from sensitization training so that they can better support their clients and their menstrual needs.

Overwhelmingly, the largest barrier to the suggestions proposed is lack of funding; however, compared to many city-funded initiatives, these costs would be minimal. We fund what we care about, and to date, addressing the menstrual needs of vulnerable populations has been a low priority. Efforts such as the *NYC Menstrual Policy* show positive progress, but have not yet achieved their goal of ensuring menstrual product access. At the national level, there has also been some progress. A growing number of cities and states have passed policies mandating the distribution of free pads in public schools, homeless shelters and correctional facilities. Additionally, during the COVID-19 pandemic, the USA government classified pads and tampons for the first time as medical expenses that are eligible for flexible spending accounts (FSA) and health saving account (HSA) reimbursement in the Coronavirus Aid, Relief and Economic Securities (CARES) Act (35). While this will only support certain segments of the USA population, it is an important recognition that menstrual products are essential items.

### Limitations

There are some limitations important to note. First, the study sample was small, and although it included participants with experience living on the street and in shelters, it was predominantly those living in shelters. Similarly, while our study was open to all individuals with concurrent experiences of being homeless and menstruating, all of our study participants were cisgender women. It is likely that individuals who menstruate and do not identify or present as female face increased challenges to accessing menstrual products, as was also noted by a number of key informants. A larger sample would have likely captured a broader range of experiences, challenges and successes around accessing menstrual products while experiencing homelessness. Second, our sample was limited to individuals who were capable of providing informed consent. As a result, the study excluded individuals struggling with mental health conditions, who represent a sizeable portion of the homeless population, and likely face even more significant barriers to managing their menstruation.

## Conclusion

Overall, this study illustrated how individuals experiencing homelessness in NYC, whether living in shelters or on the street, are often not able to access the menstrual products that they need to manage their monthly menstrual flow. While the 2016 *NYC Menstrual Policy* represented an important step towards ensuring this population can access menstrual products, as yet it does not assure consistent and adequate access to these basic necessities. Although additional research is needed to better understand the menstrual experiences and needs of a broader range of individuals experiencing homelessness, our findings suggest a few actionable items that could improve menstrual product access in NYC. These include increasing free product distribution in locations more widely accessible to individuals living on the street; training shelter and service provider staff on menstruation to improve their comfort around providing these services; and rethinking distribution strategies in shelters and at other service providers. This study also underscores the critical importance of expanding efforts that seek to provide access to these essential items for vulnerable populations across the country.

## Data Availability

The dataset used (all transcripts) and analysed during the current study are available from the corresponding author on reasonable request.

## Abbreviations

CARES: Coronavirus Aid, Relief and Economic Securities
DCAS: Department of Citywide Administrative Services
FSA: Flexible Spending Account
HSA: Health Spending Account
HUD: Department of Housing and Urban Development
IDI: In-depth interview
IRB: Institutional Review Board
KII: Key informant interview
NYC: New York City
SNAP: Supplemental Nutrition Assistance Program
USA: United States of America
WIC: Special Supplemental Nutrition Program for Women, Infants, and Children
